# Contextual modulation of sensitivity to naturalistic image structure in macaque V2

**DOI:** 10.1152/jn.00900.2017

**Published:** 2018-04-11

**Authors:** Corey M. Ziemba, Jeremy Freeman, Eero P. Simoncelli, J. Anthony Movshon

**Affiliations:** ^1^Center for Neural Science, New York University, New York, New York; ^2^Howard Hughes Medical Institute, New York University, New York, New York

**Keywords:** natural image statistics, surround suppression, texture, V2, visual cortex

## Abstract

The stimulus selectivity of neurons in V1 is well known, as is the finding that their responses can be affected by visual input to areas outside of the classical receptive field. Less well understood are the ways selectivity is modified as signals propagate to visual areas beyond V1, such as V2. We recently proposed a role for V2 neurons in representing the higher order statistical dependencies found in images of naturally occurring visual texture. V2 neurons, but not V1 neurons, respond more vigorously to “naturalistic” images that contain these dependencies than to “noise” images that lack them. In this work, we examine the dependency of these effects on stimulus size. For most V2 neurons, the preference for naturalistic over noise stimuli was modest when presented in small patches and gradually strengthened with increasing size, suggesting that the mechanisms responsible for this enhanced sensitivity operate over regions of the visual field that are larger than the classical receptive field. Indeed, we found that surround suppression was stronger for noise than for naturalistic stimuli and that the preference for large naturalistic stimuli developed over a delayed time course consistent with lateral or feedback connections. These findings are compatible with a spatially broad facilitatory mechanism that is absent in V1 and suggest that a distinct role for the receptive field surround emerges in V2 along with sensitivity for more complex image structure.

**NEW & NOTEWORTHY** The responses of neurons in visual cortex are often affected by visual input delivered to regions of the visual field outside of the conventionally defined receptive field, but the significance of such contextual modulations are not well understood outside of area V1. We studied the importance of regions beyond the receptive field in establishing a novel form of selectivity for the statistical dependencies contained in natural visual textures that first emerges in area V2.

## INTRODUCTION

Spatial vision relies on neurons in a sequence of brain areas that transform their inputs to extract progressively more complex visual information. Neurons in the primary visual cortex (V1) combine inputs from multiple neurons in the lateral geniculate nucleus (LGN), resulting in V1 receptive fields that are both larger and more selective to stimulus orientation. Similarly, V2 neurons combine the responses of multiple V1 neurons, resulting in receptive fields that are about twice the diameter of those found in V1 (Gattass et al. 1981; Shushruth et al. 2009). However, this increase in receptive field size is not accompanied by substantial changes in selectivity to stimuli typically used for V1 receptive field characterization (such as drifting bars or sinusoidal gratings).

The spatial region of sensitivity of a neuron resulting from this feedforward pooling is often referred to as the “classical receptive field” (CRF), but the responses of most visual neurons are also affected by image content outside of this region. Such effects are thought to be mediated by recurrent and feedback connections (Angelucci et al. 2002; Cavanaugh et al. 2002a). These contextual influences have been extensively studied in V1 and generally act to suppress responses (Blakemore and Tobin 1972; Cavanaugh et al. 2002a; DeAngelis et al. 1994; Levitt and Lund 1997; Sceniak et al. 1999). The strength of this “suppressive surround” in V1 is dependent on many stimulus attributes, including orientation, spatial frequency, and spatial proximity (Cavanaugh et al. 2002b). However, influences from outside the CRF are generally believed to play a modulatory role, rather than a direct role in establishing selectivity for visual features more complex than those represented within the CRF (but see Dobbins et al. 1987; Gilbert and Wiesel 1990; Hallum and Movshon 2014). Although there is some evidence for categorically different surround properties between V1 and V2 neurons responding to binocular and chromatic stimuli (Solomon et al. 2004; Thomas et al. 2002), they are quite similar when examined with drifting gratings (Shushruth et al. 2009), and the effects of more complex spatial patterns have not been explored.

Natural images contain orderly structures that drive correlations in the output of V1 neurons tuned to different positions, orientations, and spatial frequencies. Surround mechanisms have been proposed to increase the efficiency of information transmission by reducing naturally occurring dependencies between feedforward V1 responses (Coen-Cagli et al. 2015; Schwartz and Simoncelli 2001; Vinje and Gallant 2002). However, we recently found that the joint activity induced in V1 by the structure of natural images may underlie a form of V2 neuronal selectivity that is absent in V1 (Freeman et al. 2013; Ziemba et al. 2016). We generated synthetic texture images that either contained or lacked the statistical dependencies in simulated V1 output found in natural images. V2 neurons, but not V1 neurons, fired more vigorously to the presentation of large, naturalistic textures than to spectrally matched “noise” stimuli that lacked these higher order statistical dependencies. Reducing the diameter of the stimuli to match the estimated CRF diminished this effect in many V2 neurons, suggesting that stimulation of the surround might help to create sensitivity to naturalistic image structure (Freeman et al. 2013).

In this article, we report the results of size tuning experiments in a population of V2 neurons using both naturalistic textures and spectrally matched noise. Generally, responses were suppressed when stimuli extended beyond the CRF while also becoming increasingly sensitive to naturalistic image structure. Part of this enhanced sensitivity arose from weaker surround suppression for naturalistic than for noise stimuli. Furthermore, the dynamics of enhanced naturalistic sensitivity in V2 mirrored the time course of surround suppression, suggesting a similar origin in lateral interactions or feedback connections. We conclude that the receptive field surround of V2 neurons plays a role in establishing selectivity for the joint activity patterns of V1 neurons driven by natural images.

## MATERIALS AND METHODS

### 

#### Visual stimuli.

We generated synthetic texture stimuli using the analysis-synthesis procedure described by Portilla and Simoncelli (2000; see endnote regarding available software and examples). We measured the statistics of 32 grayscale photographs (the same ones used to generate stimuli for single-unit physiology in Freeman et al. 2013). Each photograph had a resolution of 320 × 320 pixels and served as the prototype for a texture “family.” Each image was decomposed using a steerable pyramid, which uses a bank of filters with four orientations and four spatial scales that tile the Fourier domain and constitute an invertible linear transform. For each filter, we computed the linear responses as well as the local magnitude responses (square root of sum of squared responses of the filter and its Hilbert transform), roughly analogous to the feedforward responses of V1 simple and complex cells, respectively. We then computed pairwise products across filter responses at different positions (within each orientation and scale and across a 7 × 7 neighborhood) for both sets of responses and (for the magnitudes only) across different orientations and scales. We also included products of linear filter responses with phase-doubled responses at the next coarsest scale (see Portilla and Simoncelli 2000 for a more detailed description of all parameters). All pairwise products were averaged across the spatial extent of the image, yielding correlations. The correlations of the linear responses are second-order statistics, in that they represent the averages of quadratic functions of pixel values. The correlations of magnitudes (and phase-doubled responses) are of higher order because of the additional nonlinearities in the magnitude (and phase doubling) computation. We additionally computed the average magnitude within each frequency band and the third- and fourth-order marginal pixel statistics (equivalently, the skew and kurtosis).

We generated synthetic textures for each family by initializing with an image of Gaussian white noise and adjusting it until it matched the model parameters computed on the corresponding original image (Portilla and Simoncelli 2000). We also generated spectrally matched noise images for each family by randomizing the phase but matching the complete two-dimensional power spectra of the synthetic texture images. For all experiments, we presented images at the same scale (80 pixels/degree) as in Freeman et al. (2013) but windowed them with a variable-diameter aperture. When performing the size tuning experiments, we changed the aperture diameter but maintained the same scale. We presented 15 different “samples” of both naturalistic and spectrally matched noise for each texture family, each initialized with a different noise seed.

#### Neurophysiology.

We recorded isolated single units in area V2 from three anesthetized, paralyzed adult macaque monkeys (*Macaca fascicularis*). Our standard methods for surgical preparation are given in detail elsewhere (Cavanaugh et al. 2002a). We maintained anesthesia with infusion of sufentanil citrate (6–30 μg·kg^−1^·h^−1^) and paralysis with infusion of vecuronium bromide (Norcuron; 0.1 mg·kg^−1^·h^−1^) in isotonic dextrose-Normosol solution. We monitored vital signs (heart rate, lung pressure, EEG, body temperature, urine volume and specific gravity, and end-tidal Pco_2_) and maintained them within the appropriate physiological range. The eyes were protected with gas-permeable contact lenses; supplementary lenses chosen through direct ophthalmoscopy made the retinas conjugate with a screen 114 cm distant. At the conclusion of data collection, the animal was killed with an overdose of pentobarbital sodium. All experimental procedures were conducted in compliance with the NIH *Guide for the Care and Use of Laboratory Animals* and with the approval of the New York University Animal Welfare Committee. We made a craniotomy and durotomy centered ~2–4 mm posterior to the lunate sulcus and 10–16 mm lateral, and individually advanced several quartz-platinum-tungsten microelectrodes (Thomas Recording) into the brain in a parasaggital plane at an angle 20° from vertical. We presented visual stimuli on a gamma-corrected cathode ray tube monitor (Eizo T966; mean luminance 33 cd/m^2^) at a resolution of 1,280 × 960 with a refresh rate of 120 Hz. For each isolated unit, we first determined its ocular dominance and occluded the nonpreferred eye.

#### Experimental procedure.

We made extracellular recordings from every single unit with a spike waveform that rose sufficiently above noise to be reliably isolated, and we fully characterized every unit that demonstrated a measurable visually evoked response to gratings or naturalistic texture stimuli. We made no attempt to target any particular layer of cortex, and our results represent a roughly uniform sampling from different cortical depths. Because different V2 units are selective for different higher order statistics (Freeman et al. 2013; Okazawa et al. 2017), we aimed to characterize the size tuning in response to texture families tailored to each unit (note that a full set of measurements for all texture families would have required more than 6 h of recording time). For each neuron, we chose 1–6 texture families based on the strength of the modulation index (difference divided by sum of responses to naturalistic and noise stimuli) measured within a 4° aperture, regardless of the sign, although in practice the largest modulation indexes were nearly always positive. Data are reported from V2 units for which we were able to measure (with 7 or more repetitions of each sample) both naturalistic and noise stimuli within apertures of all sizes. This minimum characterization required roughly 1.5 h of recording time per unit. In total, we completed 111 size-tuning experiments across 42 different V2 neurons.

#### Analysis.

We calculated the response for all analyses as the firing rate within a time window matched to the duration of the stimulus, shifted by the latency of each neuron. For the size tuning data from drifting gratings, we fit a ratio of Gaussians model as described in Cavanaugh et al. (2002a) to the responses of each neuron responding to both circular and annular patches. We then took twice the standard deviation of the excitatory Gaussian to be our estimate of the CRF diameter. We substituted the optimal size of the fitted model response as the CRF estimate for a small number of neurons (6) exhibiting strong surround suppression, which drove unrealistic estimates of center size. For subsequent analyses, we used these CRF estimates because drifting gratings usually evoked the largest spike rates at optimal sizes, and also so as to use an independent estimate of CRF size not influenced by the texture statistics. Results were qualitatively similar, however, when we used estimates from the responses to either naturalistic or noise stimuli. The modulation index values we report were computed from responses to the largest stimulus shown to each neuron, except where otherwise noted.

We estimated the response latency of each neuron by maximizing the stimulus-associated response variance (Smith et al. 2005). Specifically, this procedure finds the 100-ms time window position that maximizes the variance of the mean firing rate across different stimulus conditions. We aligned each neuron to its estimated response onset and binned spikes for each experiment at 1 ms, smoothing the peristimulus time histogram (PSTH) with a causal exponential filter with a time constant of 10 ms and averaging the traces from all experiments together. We present the data as a function of response rather than stimulus onset to more precisely demonstrate the delayed emergence of modulatory effects. The time course of suppression and modulation indexes were computed separately for each experiment on the raw spike counts, smoothed with the same exponential filter, and then averaged together. To capture the dynamics of suppression we divided the difference of maximum and suppressed responses by their sum to increase the stability of the index for low spike counts and to make a direct comparison to the modulation index, which is calculated in the same way. We averaged conditions together with relative sizes ranging from 0.8 to 1.3 times the CRF (mean = 1.0) for the CRF-matched condition and greater than 3.5 times the CRF (mean = 6.4) for the large stimulus condition. We performed the same procedure when analyzing annular stimuli, but with relative inner diameters between the bin edges used for the CRF-matched and large-stimulus conditions (1.3–3.5 times the CRF; mean = 2.1).

To examine parameter stability with stimulus size, we first measured parameters of the Portilla-Simoncelli texture model (Portilla and Simoncelli 2000) from 15 samples of naturalistic and spectrally matched noise stimuli drawn from all 32 texture families used in our initial neural characterization. We separated out parameter groups, as we have done previously (Freeman et al. 2013). In the present study, we focused on magnitude correlations across scale (48 parameters), position (384 parameters), and orientation (24 parameters) because they seem to be most important for establishing sensitivity to naturalistic visual structure (Freeman et al. 2013; Okazawa et al. 2015, 2017; Ziemba et al. 2016). We measured each sample at multiple image sizes by cropping the full image down to dimensions ranging from 64 × 64 to 704 × 704 pixels (equivalent to 0.8°–8.8° of visual angle). For each texture family and image size, we subtracted the parameter value for one sample of spectrally matched noise from that measured for one sample of naturalistic stimuli and divided by their sum, analogous to the computation of the modulation index we used for physiology. We computed the mean and variance of this parameter modulation index across each combination of samples of spectrally matched noise and naturalistic texture from a particular family, and we averaged these values across all 456 parameters and 32 texture families to get our final measure as a function of image size.

## RESULTS

**Fig. 1.**
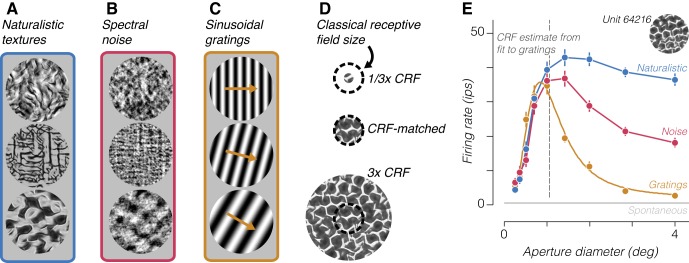
Example stimuli and neuronal tuning. *A* and *B*: 3 examples of naturalistic textures from different families (*A*) and the corresponding spectrally matched noise stimuli (*B*). *C*: 3 examples of drifting sinusoidal gratings of different orientation and spatial frequency used to characterize receptive fields. *D*: design of the size tuning experiment. The classical receptive field (CRF) size of an example V2 neuron is represented as a dashed circle. Stimuli were centered on the receptive field and presented through an aperture of varying size. *E*: example size tuning curves measured from a single V2 neuron stimulated by 3 different types of stimuli. Yellow circles represent the measured average firing rate to drifting gratings of different diameter, and yellow curve represents the fitted ratio of Gaussians model from which we derive an estimate of the CRF size. Blue trace represents the average firing rate to different samples of the naturalistic texture family indicated by *inset* image, and red trace represents the average firing rate to samples of the corresponding spectrally matched noise. Error bars represent ±SE across samples and repetitions. Gray horizontal line represents spontaneous firing rate, and dashed vertical line represents the CRF size estimate. ips, Impulses/s.

We generated synthetic stimuli with naturally occurring marginal and joint statistics across the outputs of a simulated population of V1 simple and complex cells (Portilla and Simoncelli 2000). We refer to images generated using the full set of parameters as naturalistic (Fig. 1*A*). We additionally created spectrally matched noise stimuli by randomizing the Fourier phases of each naturalistic image (Fig. 1*B*). These noise stimuli contain the same average orientation and spatial frequency content, but they do not preserve the higher order statistics of the naturalistic stimuli (see materials and methods). These two image types evoke equal firing rates in V1 neurons, on average, whereas V2 neurons are driven more strongly by naturalistic stimuli (Freeman et al. 2013).

We recorded responses from 42 V2 neurons in three anesthetized macaque monkeys to sequences of naturalistic and spectrally matched noise stimuli. For each neuron, we first characterized its CRF using drifting sinusoidal gratings (Fig. 1*C*). From the response to drifting gratings of various sizes, we derived an estimate of the CRF diameter for use in analyzing responses to texture stimuli. We subsequently measured responses to naturalistic and spectrally matched noise images presented within a 4° aperture centered on the receptive field (Freeman et al. 2013). We presented 15 “samples” of both naturalistic and noise stimuli drawn from 32 different texture “families” synthesized from 32 original images. One naturalistic and one spectrally matched noise sample from three families are shown in Fig. 1, *A* and *B*, respectively. We presented each image for 100 ms, followed by 100 ms of mean luminance. We computed a modulation index from the responses to each texture family by subtracting the firing rates to naturalistic and spectrally matched noise samples and dividing by their sum. After this initial characterization, we chose a number of texture families for additional characterization based on the strength of this modulation index. For each chosen texture family, we performed a size tuning experiment by varying the diameter of the aperture of the texture patch in logarithmically spaced intervals centered around our online estimate of the neuron’s CRF size (Fig. 1*D*). Importantly, we varied the size of our texture stimuli by masking the full images, and not by rescaling them. The image content in the center of the receptive field was therefore identical for large and small size conditions (Fig. 1*D*). A typical example of size tuning curves measured from a single V2 neuron responding to drifting gratings and naturalistic and spectrally matched noise samples from a particular texture family are shown in Fig. 1*E*.

**Fig. 2.**
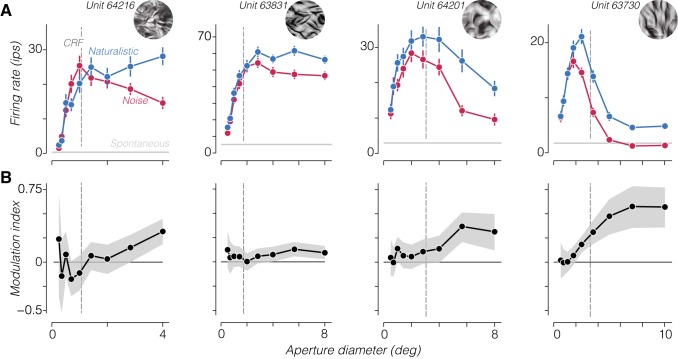
Size dependence of naturalistic sensitivity. *A*: average firing rate to naturalistic and noise stimuli from a particular family for 4 example V2 neurons as a function of the aperture diameter of the stimulus. Error bars are SE across samples and repetitions. Gray horizontal lines represent mean spontaneous activity for each neuron. Dashed vertical line represents classical receptive field (CRF) size estimated from responses to drifting gratings. *Inset* images show an example of the texture family used to stimulate each neuron. Panel at *far left* shows a tuning curve derived from responses to a second texture family from the same neuron shown in Fig. 1*E*. *B*: modulation index (difference divided by sum) computed from the firing rates in *A*. Shaded regions represent 95% confidence intervals. ips, Impulses/s.

We found a wide range of receptive field sizes and size-tuning shapes across our recorded population (Fig. 2*A*). There were clear differences between size tuning to naturalistic and spectrally matched noise stimuli for most neurons. Specifically, these stimuli evoked similar responses at small sizes, whereas a preference for naturalistic textures emerged gradually as the size of the stimulus increased. We quantified this by computing a modulation index for each size (Fig. 2*B*). Modulation strength increased with aperture diameter and continued to increase for sizes well beyond the CRF for most neurons.

**Fig. 3.**
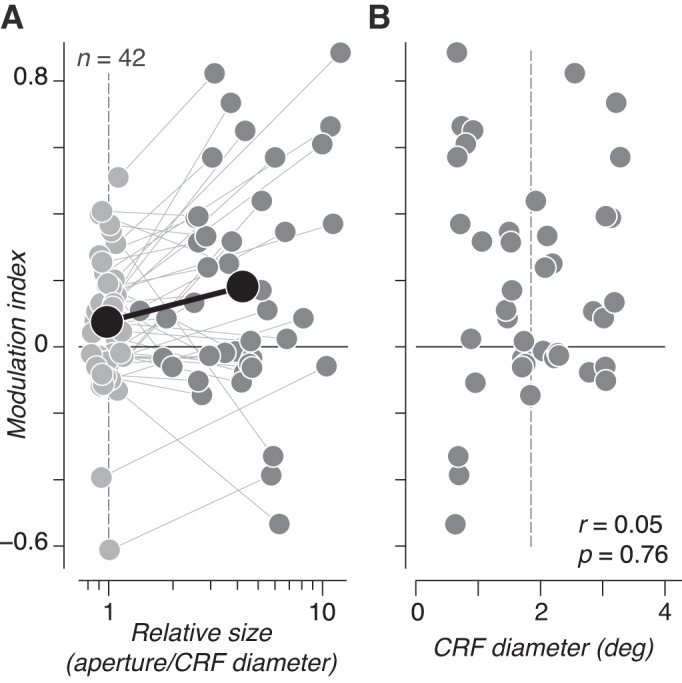
Population summary of size dependence of naturalistic sensitivity. *A*: modulation index measured in response to stimuli matched to classical receptive field (CRF) size (light shaded circles) and from the largest stimulus shown (dark shaded circles) is plotted for each V2 neuron. Lines connect measurements from the same neuron. The population-averaged modulation index and relative size are plotted in black. Modulation indexes measured at both sizes were significantly >0 (*P* < 0.05, *t*-test), and modulation indexes were significantly larger for large vs. CRF-matched sizes (*P* < 0.05, paired *t*-test). *B*: estimated CRF diameter vs. modulation index measured from the largest stimulus shown to each neuron. There was no evidence of a relationship between CRF diameter and modulation index. Dashed vertical line in *A* and *B* represents the average CRF diameter across the population.

To analyze this trend across the population, we divided the aperture diameter of the stimuli presented to each neuron by the diameter of that neuron’s estimated CRF. We then examined the modulation index computed from responses to the stimulus size closest to its CRF diameter and from responses to the largest stimulus shown (Fig. 3*A*). For CRF-matched sizes, most V2 neurons showed a small but reliable modulation index (Fig. 3*A*, light shaded circles; 0.08 ± 0.03, mean ± SE across neurons; *P* < 0.05, *t*-test). At the largest sizes we measured, modulation strength across the population more than doubled compared with that for CRF-matched stimuli (Fig. 3*A*, dark shaded circles; 0.18 ± 0.05, mean ± SE across neurons; *P* < 0.05, paired *t*-test). Not all V2 neurons preferred naturalistic over noise stimuli or increased their preference when stimuli were larger. However, as in previous experiments (Freeman et al. 2013), this variation was not predicted by estimated CRF diameter (Fig. 3*B*; *r* = 0.05; *P* = 0.76). Instead, these results suggests that areas outside of the CRF may enhance the sensitivity of V2 neurons to naturalistic image structure (Freeman et al. 2013).

**Fig. 4.**
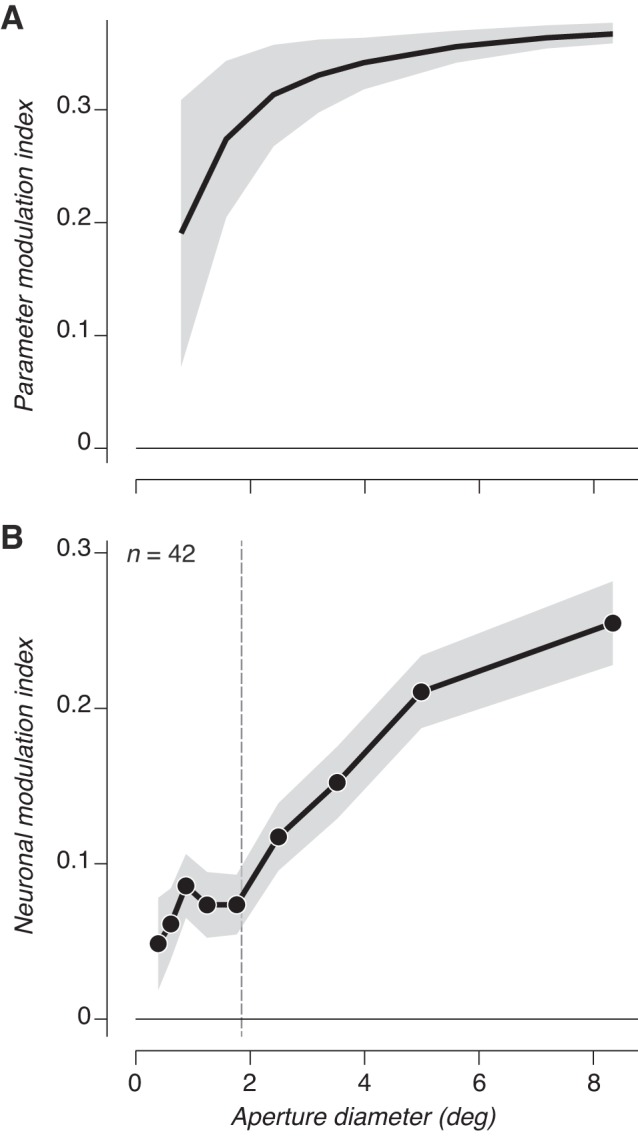
Stimulus statistics do not account for changes in sensitivity with size. *A*: parameter modulation index between naturalistic and spectrally matched noise stimuli computed from higher order parameters as a function of stimulus aperture. Shaded regions indicate average variance across samples. *B*: average neuronal modulation index from V2 responses as a function of stimulus aperture. The average classical receptive field (CRF) size is plotted as a dashed vertical line, but CRF sizes were ignored in computing the average modulation index. Shaded region represents ±SE across conditions.

We wondered whether the increase of modulation index with aperture diameter could be explained by the stimuli themselves, given that higher order statistics computed from each image depend on aperture size. Our synthesis only guarantees statistics to be fully converged to their specified values when averaged over the entire image. To examine the influence of parameter stability on neuronal responses, we analyzed the higher order statistics of samples of texture stimuli cropped at varying sizes. We focused on parameters capturing magnitude correlations across scale, position, and orientation because these have been most associated with driving sensitivity to naturalistic image structure (Freeman et al. 2013; Okazawa et al. 2015, 2017; Ziemba et al. 2016). We first measured the value of each individual parameter to samples of naturalistic and spectrally matched noise. We then computed a parameter modulation index, analogous to that used for neuronal responses, by taking the difference of each parameter value for naturalistic and spectrally matched noise stimuli and dividing by the sum (Fig. 4*A*). The strength of this parameter modulation index was biased toward lower values and highly variable when the image was small, but it approached an asymptotic value once stimuli reached ~2°, close to the average receptive field size of our V2 population (1.7 °± 0.8°, mean ± SD; Fig. 4*B*).

To compare directly with physiology, we took all size conditions across all experiments in all units and ordered the modulation indexes and stimulus diameter vectors by stimulus diameter and then averaged all values together in bins of 111 observations. This procedure gave us the average neuronal modulation index as a function of absolute size (disregarding individual CRF sizes). This modulation index more than doubled as the stimulus grew from 2° to 8°, differing markedly from the parameter modulation index. Although the parameter modulation index was mostly flat beyond 2°, it did increase somewhat and could potentially contribute to the increased sensitivity to naturalistic stimuli for larger stimulus sizes. However, if the bias in parameters at small sizes were a major factor in neuronal modulation, one would expect to find a correlation between CRF diameter and neuronal modulation (because V2 neurons with a small CRF should be biased toward lower modulation). We found no such correlation between CRF diameter and modulation index measured from either large (Fig. 3*B*) or CRF-matched stimuli (*r* = 0.23; *P* = 0.14). Instead, the inconsistency between stimulus size and the parameter and neuronal modulation index suggests that physiological mechanisms operating beyond the receptive field are responsible for the enhanced naturalistic sensitivity.

**Fig. 5.**
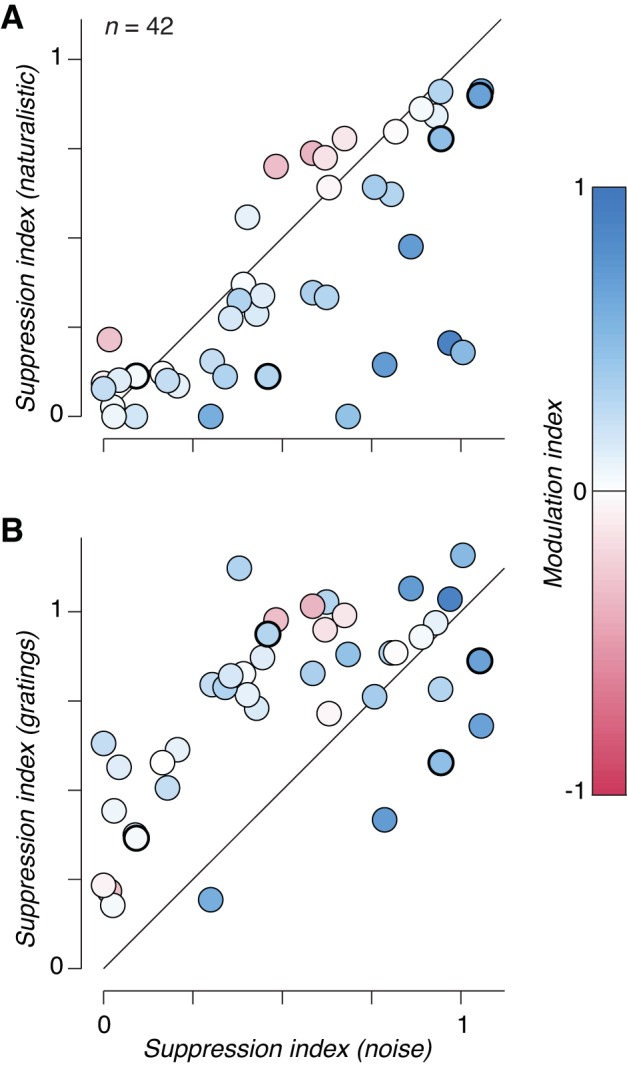
Surround suppression strength depends on naturalistic statistics. *A*: surround suppression index (fractional reduction in firing rate to large stimuli, see materials and methods) measured from responses to spectrally matched noise compared with that measured from responses to naturalistic stimuli. Each circle represents the suppression index for a neuron computed from the average responses to all families tested with size-varying apertures. Circles are colored according to the modulation index measured for the largest size stimulus. Suppression was significantly stronger in response to noise than to naturalistic stimuli (*P* < 0.005, paired *t*-test). Circles surrounded by a thick black line indicate values averaged across families for the 4 neurons depicted in Fig. 2. *B*: surround suppression index measured from responses to spectrally matched noise compared with that measured from responses to full-contrast drifting sinusoidal gratings. Circles are colored as in *A*. Suppression was significantly stronger in response to gratings compared with noise stimuli (*P* < 0.001, paired *t*-test).

What amplifies neuronal modulation as stimuli grow beyond the CRF? Typically, content outside the receptive field is suppressive (Blakemore and Tobin 1972; Cavanaugh et al. 2002a; DeAngelis et al. 1994; Levitt and Lund 1997; Sceniak et al. 1999). Surround suppression strength measured with drifting grating stimuli did not predict the strength of modulation in these or our previous experiments (*r* = 0.12; *P* = 0.44; Freeman et al. 2013). We still wondered whether suppression might play a role for more complex stimuli. As shown in Fig. 2, V2 neurons exhibit a wide range of surround suppression for both naturalistic and noise stimuli, from very little or no suppression (Fig. 2*A*, *left* 2 panels) to nearly complete suppression (Fig. 2*A*, *right*). For each neuron, we computed a suppression index from the maximum mean firing rate and the mean firing rate to the largest stimulus we presented. We subtracted the response to the largest stimulus from the maximum and divided by the maximum to obtain the fractional reduction (Cavanaugh et al. 2002a). When we compared the strength of surround suppression for naturalistic and spectrally matched noise stimuli, we found that there was significantly more suppression to noise stimuli across the population (Fig. 5*A*; naturalistic suppression index = 0.38 ± 0.05, mean ± SE; noise suppression index = 0.51 ± 0.05; *P* < 0.005, paired *t*-test). Although there was no significant relationship between the modulation index and naturalistic surround suppression (*r* = −0.1; *P* = 0.52), the correlation with noise surround suppression was significant (*r* = 0.48; *P* < 0.005). Unsurprisingly, the most modulated V2 neurons tended to be those with the largest difference in suppression (Fig. 5*A*), and the difference in suppression was a strong predictor of modulation (*r* = 0.79; *P* < 0.001). This strong correlation is expected, however, because the computation of both quantities involves subtracting the response to large noise stimuli from the response to large naturalistic textures. Furthermore, modulation was robust for many neurons with little or no surround suppression to either stimulus category (Fig. 5*A*; see Fig. 2*A*, *left* 2 panels).

**Fig. 6.**
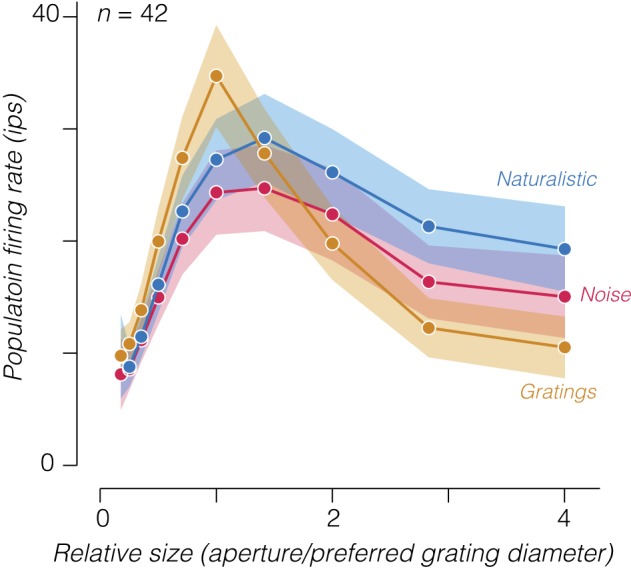
Population size tuning curves for gratings, noise, and naturalistic stimuli. We averaged together the responses of all V2 neurons by first aligning tuning curves to the stimulus aperture that drove the largest response to drifting gratings. Measurements are shown for all relative sizes that included an average over at least 30 neurons. Shaded regions represent ±SE across neurons; ips, impulses/s.

We also found that surround suppression in response to drifting gratings was significantly stronger than to either naturalistic or spectrally matched noise textures (Fig. 5*B*; grating suppression index = 0.74 ± 0.04, mean ± SE; *P* < 0.001, paired *t*-test). This is unsurprising because our texture and grating stimuli are not matched in any way and we presented grating stimuli at full contrast. High-contrast stimuli generally drive stronger surround suppression than low-contrast stimuli (Cavanaugh et al. 2002a). Gratings drove slightly higher maximum firing rates than textures across the population (Fig. 6; naturalistic = 31 ± 3.9, noise = 28 ± 3.9, and gratings = 35 ± 4.5 impulse/s, means ± SE across neurons), dissociating the strength of surround suppression from the strength of response to an optimally sized stimulus across the different stimulus types. At large sizes, naturalistic stimuli drove the highest firing rate, followed by noise, and then gratings (Fig. 6; naturalistic = 21 ± 3.6, noise = 17 ± 3.7, and gratings = 12 ± 2.4 impulses/s, mean ± SE across neurons). This indicates that surround suppression strength, and thus the firing rate to large stimuli, roughly follows the “naturalness” of the stimuli. A grating contains only a single orientation and spatial frequency, whereas our noise stimuli contain the power spectrum of natural images and our naturalistic stimuli contain further higher order statistics contained in natural images. The difference in surround suppression between gratings and textures is likely related to observations in V1 where content outside the receptive field is maximally suppressive when it is matched for features (e.g., orientation) presented to the center (Cavanaugh et al. 2002a; Sillito et al. 1995). However, the difference in surround suppression between naturalistic and noise textures represents a novel observation.

**Fig. 7.**
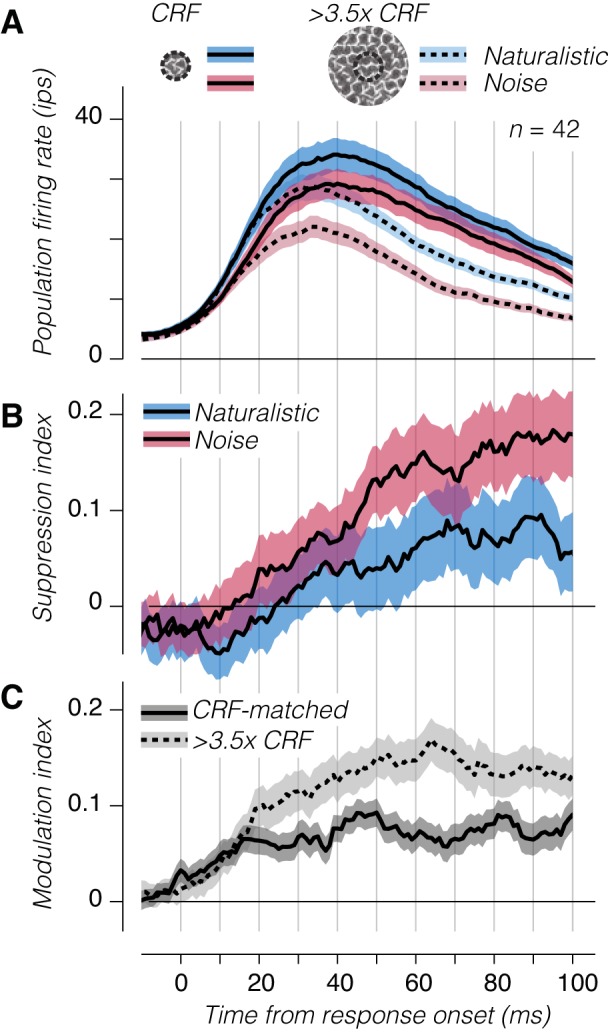
Temporal dynamics of suppression and sensitivity across the V2 population. *A*: average population firing rate of V2 neurons to large and small patches of naturalistic or spectrally matched noise. The response of each neuron was aligned to its estimated response onset. *B*: normalized suppression as a function of time from response onset for naturalistic and spectrally matched stimuli. *C*: modulation index as a function of time for classical receptive field (CRF)-matched and large stimuli. Shaded regions represent ±SE across experiments; ips, impulses/s.

To further elucidate the relationship between surround suppression and the strength of naturalistic modulation in V2, we examined the time course of responses for these two effects. We gathered trials with stimulus diameters approximately equal to that of the CRF, as well as those with diameter greater than three and a half times the CRF, aligned the recordings of individual neurons to their estimated response onset latency, and computed the average firing rate as a function of time (Fig. 7*A*). These traces show an earlier separation in firing rates between responses to naturalistic and noise stimuli (at either stimulus size) than between CRF-matched and larger stimuli (Fig. 7*A*).

We examined the dynamics of suppression in more detail by computing the surround suppression index at each time point (Fig. 7*B*). As has been previously observed (Bair et al. 2003; Henry et al. 2013; Shapley et al. 2007; Webb et al. 2005), suppression from the receptive field surround was delayed, beginning to affect responses ~20 ms after response onset for noise stimuli and around 10 ms later for naturalistic stimuli (Fig. 7*B*). Surround suppression to spectrally matched noise also reached a higher value, consistent with our results when aggregating spikes across the entire stimulus window (Fig. 5*A*). Delays in the onset of surround suppression have been interpreted to reflect the involvement of lateral interactions or feedback connections from higher visual areas (Angelucci et al. 2002; Cavanaugh et al. 2002a).

The temporal dynamics of the modulation index had a different but related temporal profile (Fig. 7*C*). For the CRF-matched condition, modulation began to rise at response onset and reached its steady-state level around 15 ms later. When the stimulus was large, however, the modulation index continued to rise, surpassing the modulation strength for CRF-matched stimuli around 10–20 ms after response onset. This spatiotemporal profile indicates that whereas one component of sensitivity to naturalistic texture arises rapidly from CRF stimulation, another component emerges with a delayed time course resembling that of surround suppression, suggesting the possibility of a shared origin in recurrent or feedback interactions.

**Fig. 8.**
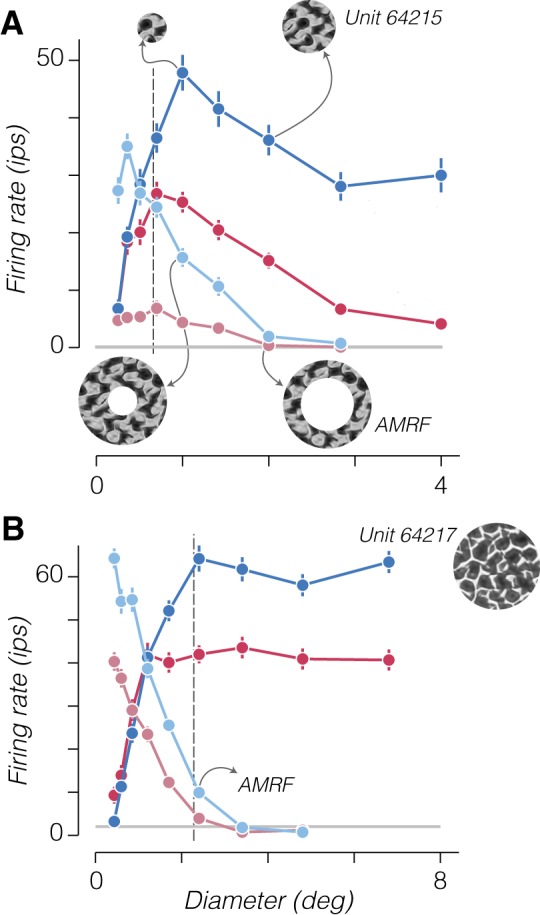
Responses to annular stimuli reveal surround facilitation to naturalistic stimuli. *A*: saturated color traces show responses of an example cell to a circular patch of noise (red) or naturalistic (blue) texture. Desaturated color traces show responses to an annular patch. The *x*-axis indicates the outer diameter of circular patches and inner diameter of annular patches. *Inset* images show example circular (*top*) and annular (*bottom*) stimuli with matched outer and inner diameters, respectively. The example annular stimulus at *bottom right* represents the size of the annular minimum response field (AMRF) for the neuron, because naturalistic stimuli windowed within an annulus with this inner diameter drove a response above the spontaneous rate. Error bars represent ±SE across samples and repetitions. Gray horizontal line represents mean spontaneous activity. Dashed vertical line represents the classical receptive field size estimate from gratings. *B*: responses from a different example cell exhibiting no surround suppression and a strong preference for naturalistic over noise stimuli presented within an annulus for all sizes that drive a response. *Inset* image shows an example of the texture family used to stimulate the neuron. Arrow indicates diameter of the AMRF for this neuron; ips, impulses/s.

Stronger responses to spatially extensive naturalistic stimuli (compared with spectrally matched noise) could result from either a weaker surround suppression or an enhanced surround facilitation (with similar spatial summation and dynamics). We wondered whether we could distinguish these two scenarios by examining responses to a second set of stimuli consisting of naturalistic and noise textures windowed by an annulus. We fixed the outer diameter of these stimuli to the size of the largest circular patch used to stimulate a given neuron, and we varied the inner diameter. Figure 8*A* shows the responses of an example V2 neuron to stimuli windowed by both circular and annular apertures as a function of outer and inner diameter, respectively. Responses to annular stimuli with small inner diameters resembled the response to large circular patches and fell with increasing inner diameter as the excitatory drive to the CRF was withdrawn. The area between ~1° and 2° in Fig. 8*A* represents an annular region of the visual field where the stimulus can either be suppressive or excitatory depending, respectively, on whether the center or surround are simultaneously stimulated (Cavanaugh et al. 2002a). The outcome of surround stimulation can thus be modified through altering the stimulus drive delivered to the center, an effect captured by the phenomenon of cortical normalization (Heeger 1992; Schwartz and Simoncelli 2001).

Could the decreased suppressive influence of naturalistic stimuli in the surround arise from altering the balance of center and surround mechanisms through known normalization computations? Such a computation likely underlies the effect of contrast reduction on the summation properties of V1 neurons stimulated by drifting gratings (Cavanaugh et al. 2002a; Sceniak et al. 1999). Reducing the contrast of a circular patch of grating decreases both the drive to the center and the relative influence of the surround on responses, resulting in a net reduction in firing rate and a shift to larger optimal sizes (Cavanaugh et al. 2002a). In contrast, our results show that eliminating higher order statistics (i.e., transitioning from textures to spectrally matched noise stimuli) appeared to reduce the drive to the center but increase the relative influence of the surround. In addition, stimulation with naturalistic texture appeared to increase the optimal size for circular patches in many neurons (Fig. 2*A* and Fig. 8, *A* and *B*), as if a spatially extended facilitatory mechanism were engaged.

Neurons with little or no surround suppression allow us to test this idea; an example is shown in Fig. 8*B*. This neuron showed little preference for naturalistic texture when we presented stimuli within small circular patches but a strong preference when stimuli were presented within large patches that covered the receptive field surround (Fig. 8*B*, solid color traces). An annular stimulus with a large inner diameter evoked no response, but when the inner diameter was made smaller than 2.5°, the neuron responded weakly but with a very strong preference for naturalistic stimuli (Fig. 8*B*, shaded color traces). This indicates that naturalistic texture in the surround strongly facilitated the responses driven by weak CRF stimulation (and cannot be explained through a release from surround suppression, because this neuron exhibited none).

**Fig. 9.**
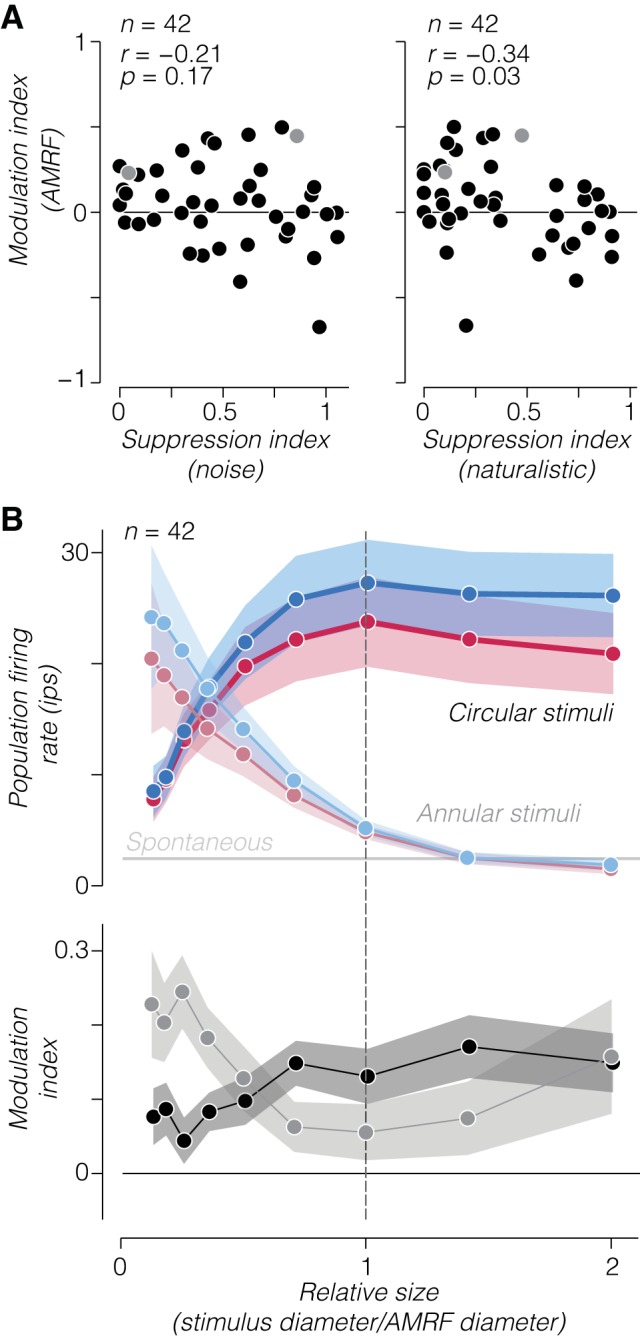
Dissociation of surround suppression strength from naturalistic sensitivity to annular stimuli. *A*: scatterplot of the modulation index measured from annular stimuli matched to the annular minimum response field (AMRF) of each V2 neuron against surround suppression to noise (*left*) and naturalistic (*right*) stimuli. Lighter shaded symbols in each panel correspond to the 2 example neurons in Fig. 8, *A* and *B*. *B*: average population firing (*top*) and modulation index (*bottom*) as a function of relative size of annulus inner diameter (light shading) and circular aperture diameter (dark shading) for all V2 neurons. Tuning curves were aligned to each neuron’s estimated AMRF. In *top* panel, gray horizontal line represents mean population spontaneous firing rate. Dashed vertical line represents the estimated AMRF. Shaded regions represent ±SE across neurons; ips, impulses/s.

To examine naturalistic sensitivity to annular stimuli across the population, we identified the largest inner diameter for each neuron that drove a response 1 SD above the spontaneous rate. We refer to this diameter as the annular minimum response field (AMRF). We then computed the modulation index measured from an annulus matched to the AMRF of each neuron. In contrast to modulation measured for a large circular stimulus, this AMRF modulation was uncorrelated with surround suppression to noise (Fig. 9*A*, *left*; *r* = −0.21; *P* = 0.17) and actually weakly anticorrelated with surround suppression to naturalistic stimuli (Fig. 9*A*, *right*; *r* = −0.34; *P* = 0.03; there also was no correlation with the difference in surround suppression between noise and naturalistic stimuli: *r* = 0.14; *P* = 0.37). Several V2 neurons with little or no surround suppression still showed a preference for naturalistic over noise stimuli even with minimal drive (Fig. 9*A*). We aligned all the annular responses to each neuron’s AMRF and plotted average population firing rate and modulation index as a function of relative size (Fig. 9*B*). The average modulation index was >0 (although not quite significantly so; mean = 0.06; *P* = 0.14, *t*-test) for responses measured from AMRF-aligned stimuli, and even for stimuli that did not drive a response above spontaneous firing (Fig. 9*B*; however, modulation indexes can become very large and unstable for low firing rates). We think it unlikely that this relatively weak average modulation explained the lack of significant correlation with surround suppression to noise stimuli, because this correlation remained nonsignificant for annular stimuli of all relative sizes (including those yielding average modulation indexes that were significantly different from 0). These results suggest that V2 neurons minimally driven by annular stimuli are likely to be facilitated by naturalistic image structure in their surround and not released from a stronger suppression to noise stimuli.

**Fig. 10.**
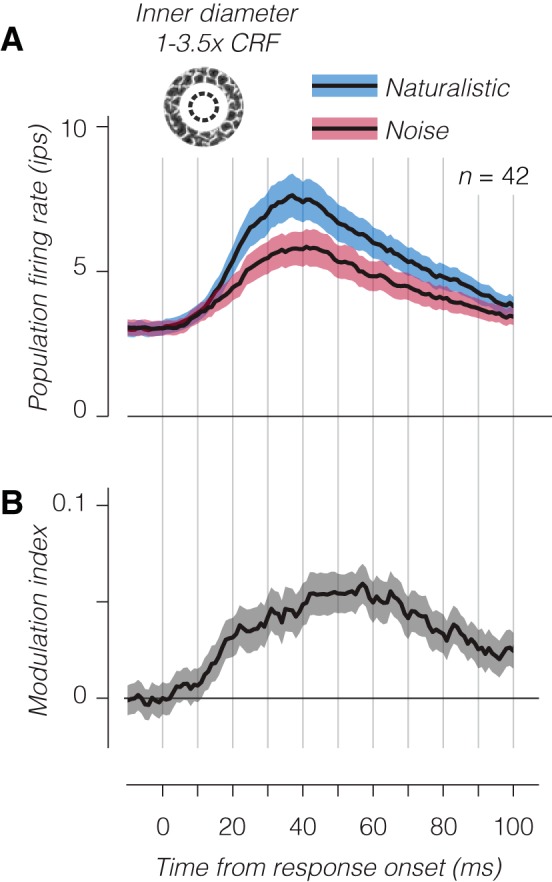
Temporal dynamics of population responses to annular stimuli. *A*: average population firing rate of V2 neurons to annular patches of texture with inner diameter between the classical receptive field (CRF) diameter and 3.5 times the CRF. As in Fig. 7, the response of each neuron was aligned to its estimated response onset before averaging. *B*: modulation index as a function of time. Shaded regions represent ±SE across experiments.

**Fig. 11.**
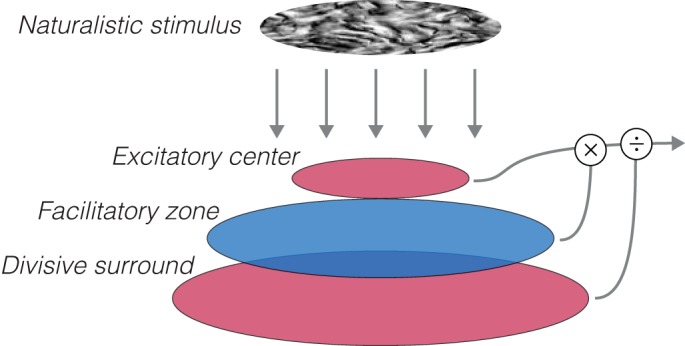
Naturalistic stimuli reveal a spatially extended facilitatory zone in V2. Cartoon schematic shows the proposed receptive field structure underlying the current results. Responses to gratings, or naturalistic or noise textures alone, can be explained through normalization mechanisms with a larger, divisive surround and center that both respond to the spectral content of the input (red). To account for the relationship between naturalistic and noise textures, a facilitatory zone larger than the receptive field center is required that is sensitive to the higher order correlations present in naturalistic textures (blue).

We further examined this effect at the population level by calculating the time course of average firing rate and modulation index to annular stimuli (Fig. 10*A*). We averaged the responses to all stimuli with inner diameter larger than the CRF (see materials and methods) and revealed a small excitatory response and a strong preference for naturalistic stimuli (Fig. 10*A*; compare with Fig. 7*A*). We also found that the emergence of the modulation index was delayed by ~10 ms for annular stimuli (Fig. 10*B*), similar to the delay before the modulation index for large stimuli surpasses the modulation index for CRF-matched stimuli (Fig. 7*C*). This small excitatory response from outside the CRF is not unique to naturalistic stimuli or V2 and is observed in V1 neurons responding to drifting gratings. However, observing a strong preference for naturalistic over noise stimuli with very weak CRF and strong surround stimulation suggests that in addition to suppression, a region surrounding the CRF of V2 neurons may facilitate responses to stimuli containing naturalistic statistical dependencies (Fig. 11), presumably through recurrent or feedback circuits.

## DISCUSSION

Neurons in area V2 exhibit distinctive sensitivity to the statistics of naturalistic textures that is absent from their V1 afferents (Freeman et al. 2013; Okazawa et al. 2017; Yu et al. 2015; Ziemba et al. 2016). In the present study, we have further dissected this enhanced sensitivity, showing that it increases as a function of stimulus size, and does so well beyond the extent of the CRF. Moreover, V2 neurons exhibit greater surround suppression for spectrally matched noise stimuli, another effect that is absent in V1 neurons. The temporal dynamics and summation properties of this surround-enhanced naturalistic texture sensitivity suggest a possible origin in long-range facilitation from recurrent circuits in V2 or feedback from higher visual areas. Although a complete account of the origin of these effects is out of reach, we can make some tentative conclusions based on previous work.

Most of our understanding of the function of receptive field surrounds comes from studying neuronal responses in V1. Stimulation of the receptive field surround suppresses responses to a degree that varies from neuron to neuron and depends on many stimulus attributes (Blakemore and Tobin 1972; Cavanaugh et al. 2002a, 2002b; DeAngelis et al. 1994; Henry et al. 2013; Levitt and Lund 1997; Sceniak et al. 1999). Most of these effects can be accounted for through divisive normalization by a surround region with tuning similar to that of the receptive field center (Cavanaugh et al. 2002a; Heeger 1992; Schwartz and Simoncelli 2001). Suppression thus acts to weaken responses when visual structure in the receptive field center and surround are redundant (Barlow 1961; Coen-Cagli et al. 2015; Schwartz and Simoncelli 2001; Vinje and Gallant 2002). These same observations and explanations in V1 appear to hold for V2 neuronal responses (Shushruth et al. 2009), perhaps because suppression is inherited from V1 or because it is produced in V2 through similar recurrent circuitry (Sincich and Horton 2005).

Our results suggest a mechanism in V2 that lies outside the CRF and is selective for naturalistic statistics. Some previous findings also suggest that surround suppression may differ between neurons in V1 and V2. V1 neurons that project to V2 tend to have stronger surround suppression (El-Shamayleh et al. 2013), and the surround may play a stronger role for V2 compared with V1 neurons in the representation of visual features such as disparity and color (Solomon et al. 2004; Thomas et al. 2002). In contrast, most studies investigating the representation of visual form find that surround suppression is similar in strength and selectivity in V1 and V2 (El-Shamayleh and Movshon 2011; Hallum and Movshon, 2014; Schmid et al. 2009, 2014; Shushruth et al. 2009; Zhang et al., 2005). Few studies of V2 have used stimuli more complex than combinations of oriented lines or gratings; however, our naturalistic stimuli may reveal details of surround suppression in V2 neurons that depend on engaging their sensitivity to higher order statistics.

Could the generally accepted framework for the suppressive surround account for our results? The naturalistic, higher order correlations in our texture stimuli yield both a stronger drive to the receptive field center and a decrease in suppression in the surround compared with spectrally matched noise (Fig. 2*A*). However, most functional accounts of surround suppression in V1 assume that the surround is driven by image features similar to those that drive the center (Ahmadian et al. 2013; Cavanaugh et al. 2002a; Schwartz and Simoncelli 2001). As such, one might predict that naturalistic textures should evoke stronger suppression than spectrally matched noise, since their content is more spatially predictable because it includes higher order statistical structure (Coen-Cagli et al. 2015). That naturalistic textures actually evoke weaker suppression could be explained if suppressive surround mechanisms in V2 had very different tuning than receptive field centers and were driven more strongly by noise than naturalistic textures. This too seems unlikely because we found that even V2 neurons with weak or no surround suppression exhibited higher sensitivity to naturalistic structure in their surrounds (Fig. 7*B*). Finally, many have suggested that suppressive surrounds in V1 could establish selectivity to complex visual form, such as curvature, and “second-order” features (Ben-Shahar and Zucker 2003; Dobbins et al. 1987; El-Shamayleh and Movshon 2011; Hallum and Movshon 2014; Tanaka and Ohzawa 2009; Walker et al. 1999). If such an explanation underlies our results, however, it is unclear why texture sensitivity would be absent in V1 (Freeman et al. 2013), where the suppressive properties of the surround appear to be broadly similar to those in V2.

Instead of surround suppression, could a spatially extended facilitation explain our results? Although nearly all V2 neurons are suppressed when image content extends outside the CRF, responses seem to be released (or facilitated) from this suppressed state when stimuli contain naturalistic structure (Fig. 11). Previous studies have suggested a facilitatory role for regions outside the CRF in V1 (Gilbert and Wiesel 1990; Li and Gilbert 2002), but perhaps more relevant here are studies that have identified differences in the strength of facilitation between V1 and V2 (Peterhans and von der Heydt 1989; Zhou et al. 2000). Signals from far outside the CRF of V2 neurons, but not V1 neurons, are thought to generate selectivity for illusory contours and figure-ground organization, or “border ownership” (Peterhans and von der Heydt 1989; Zhou et al. 2000). This selectivity is one of the few that reliably distinguish V2 from V1 neurons, but it has not been identified in anesthetized animals and may rely on top-down feedback associated with more cognitive factors such as attention and memory (Fang et al. 2009; O’Herron and von der Heydt 2013; Qiu et al. 2007). In contrast, our experiments were conducted under anesthesia and thus likely reflect a distinct form of facilitation.

What might be the source of this facilitation? The temporal dynamics of surround-enhanced naturalistic sensitivity in V2 are delayed, like those of tuned suppression in V1 (Fig. 7; Bair et al. 2003; Henry et al. 2013; Knierim and van Essen 1992; Shapley et al. 2007), which are thought to arise from a combination of long-range horizontal connections and feedback from higher visual areas (Angelucci et al. 2002). The texture-sensitive facilitatory signal in V2 could thus arise from either of these sources. Neurons in area V4 receive major V2 projections (Gattass et al. 1997; Sincich and Horton 2005), have larger receptive fields than those in V2 (Gattass et al. 1988), provide a strong feedback connection to V2 (Ungerleider et al. 2008), and have even stronger sensitivity to naturalistic statistics than V2 neurons (Okazawa et al. 2015, 2017). Facilitatory feedback from V4 may explain the effects we observed, although anesthesia would be expected to reduce the impact of such feedback.

Although our results are most consistent with a broad facilitation, more experiments are needed to solidify this interpretation (and rule out a tuned surround-suppressive mechanism). One interesting prospect is to replace the homogeneous texture patches we used with mixture stimuli containing center and surround regions with textures lacking or containing higher order statistics (or containing different higher order statistics). Use of a similar paradigm in V1 reveals that neurons responded with stronger surround suppression to full, natural images compared with mixtures in which the regions surrounding the receptive field center are phase-randomized (Coen-Cagli et al. 2015; Guo et al. 2005; Pecka et al. 2014). We created our spectrally matched noise stimuli by phase-randomizing the entire image and found that compared with naturalistic textures, this manipulation actually increased suppression in V2. Our approach using synthetic, naturalistic stimuli offers a potentially more controlled way of assessing neuronal sensitivity to variation in image statistics than partial phase randomization of natural images (which can create strong, unnatural statistics at the borders of image regions). We have preliminary evidence from experiments with mixed-texture stimuli that suggest how V2 neurons may contribute to the segmentation of regions that differ in texture (Schmid and Victor 2014; Ziemba et al. 2017).

We previously found a link between perceptual sensitivity to particular statistics and the sensitivity of populations of V2 neurons (Freeman et al. 2013). Although single neurons had idiosyncratic patterns of selectivity across texture families, the pattern of sensitivity averaged across neurons was reliable across experiments and correlated with perceptual sensitivity. This could reflect the two components of naturalistic texture sensitivity suggested by our current results. Each single V2 neuron may become sensitive to a particular pattern of V1 statistics through computations performed within its receptive field center while simultaneously being attracted to an aggregate pattern of sensitivity through a broad facilitatory mechanism. Such a mechanism would suggest that neuronal and possibly perceptual sensitivity across texture families depends on aperture size. Visual neurons are generally thought to view the world through the aperture of their receptive fields. However, our results suggest that V2 neurons are sensitive to the statistical dependencies that determine the appearance of natural visual textures through mechanisms that operate at a scale much larger than their receptive fields.

## GRANTS

This work was supported by National Eye Institute Grants EY04440 and EY022428, the Howard Hughes Medical Institute, and National Science Foundation Graduate Research fellowships awarded to C. M. Ziemba and J. Freeman.

## DISCLOSURES

No conflicts of interest, financial or otherwise, are declared by the authors.

## ENDNOTE

At the request of the authors, readers are herein alerted to the fact that additional materials related to this manuscript may be found at the institutional Web site of the authors, which at the time of publication they indicate is: www.cns.nyu.edu/~lcv/texture/. These materials are not a part of this manuscript and have not undergone peer review by the American Physiological Society (APS). APS and the journal editors take no responsibility for these materials, for the Web site address, or for any links to or from it.

## AUTHOR CONTRIBUTIONS

C.M.Z., J.F., E.P.S., and J.A.M. conceived and designed research; C.M.Z. performed experiments; C.M.Z. analyzed data; C.M.Z., J.F., E.P.S., and J.A.M. interpreted results of experiments; C.M.Z. prepared figures; C.M.Z. drafted manuscript; C.M.Z., E.P.S., and J.A.M. edited and revised manuscript; C.M.Z., J.F., E.P.S., and J.A.M. approved final version of manuscript.
